# *PROX1* Gene is Differentially Expressed in Oral Cancer and Reduces Cellular Proliferation

**DOI:** 10.1097/MD.0000000000000192

**Published:** 2014-12-02

**Authors:** Maria F.S.D. Rodrigues, Camila de Oliveira Rodini, Flávia C. de Aquino Xavier, Katiúcia B. Paiva, Patrícia Severino, Raquel A. Moyses, Rossana M. López, Rafael DeCicco, Lília A. Rocha, Marcos B. Carvalho, Eloiza H. Tajara, Fabio D. Nunes

**Affiliations:** From the Department of Estomatology (MFSDR, LAR, FDN), School of Dentistry; Department of Biochemistry (KBP), Chemistry Institute; Department of Head and Neck Surgery (RAM), School of Medicine; Department of Epidemiology (RML), Public Health; Department of Genetics and Evolutionary Biology (EHT), Institute of Biosciences, University of São Paulo; Albert Einstein Research and Education Institute (PS), Albert Einstein Israelita Hospital, Center for Experimental Research; Department of Head and Neck Surgery (RDC), Arnaldo Vieira de Carvalho Cancer Institute; Department of Head and Neck Surgery (MBC), Heliopolis Hospital Complex, São Paulo; Department of Estomatology (FCdAX), School of Dentistry, Federal University of Bahia, Salvador; Department of Histology (CdOR), School of Dentistry, University of São Paulo, Bauru; and Department of Molecular Biology (EHT), School of Medicine, São José do Rio Preto, Brazil.

## Abstract

Homeobox genes are a family of transcription factors that play a pivotal role in embryogenesis. Prospero homeobox 1 (*PROX1*) has been shown to function as a tumor suppressor gene or oncogene in various types of cancer, including oral squamous cell carcinoma (OSCC). We have previously identified *PROX1* as a downregulated gene in OSCC. The aim of this study is to clarify the underlying mechanism by which PROX1 regulates tumorigenicity of OSCC cells. *PROX1* mRNA and protein expression levels were first investigated in 40 samples of OSCC and in nontumor margins. Methylation and amplification analysis was also performed to assess the epigenetic and genetic mechanisms involved in controlling *PROX1* expression. OSCC cell line SCC9 was also transfected to stably express the *PROX1* gene. Next, SCC9-PROX1-overexpressing cells and controls were subjected to proliferation, differentiation, apoptosis, migration, and invasion assays in vitro. OSCC samples showed reduced *PROX1* expression levels compared with nontumor margins. *PROX1* amplification was associated with better overall survival. *PROX1* overexpression reduces cell proliferation and downregulates cyclin D1. *PROX1*-overexpressing cells also exhibited reduced CK18 and CK19 expression and transcriptionally altered the expression of WISP3, GATA3, NOTCH1, and E2F1. Our results suggest that *PROX1* functions as a tumor suppressor gene in oral carcinogenesis.

## Abstract 2

Supplemental Digital Content is available in the text

## INTRODUCTION

Oral squamous cell carcinoma (OSCC) is the most common malignancy of the oral cavity and a major cause of cancer morbidity and mortality worldwide.^1^ Oral carcinogenesis is a multifactorial process associated with cumulative genetic mutations that alter proto-oncogenes and tumor suppressor gene function, resulting in disturbed cellular proliferation and cell differentiation.^2^

Homeobox genes encode transcriptional factors that control cellular proliferation and differentiation during embryonic development.^3^ These genes have been aberrantly expressed in solid tumors, including OSCC.^4–8^ The prospero homeobox 1 (*PROX1*) gene encodes a nuclear transcription factor (prospero homeobox protein 1/PROX1) that plays a major role during embryonic lymphangiogenesis,^9^ differentiation of the central nervous system,^10^ lens fiber elongation,^11^ and hepatocyte migration.^12^*PROX1* gene inactivation results in abnormal cell proliferation, probably because of downregulation of cell cycle inhibitors.^13^

In human cancers, *PROX1* gene acts in a tissue-dependent manner, as a transcriptional activator or repressor, leading to variable effects on cellular proliferation and differentiation.^14^*PROX1* overexpression promotes aggressive behavior of many endothelial tumors,^15,16^ colon cancer,^15,16^ and gliomas.^17^ However, in hepatocellular carcinoma, high *PROX1* expression inhibits transforming activity and cellular proliferation and is associated with well-differentiated tumors and better prognosis.^18^ Hagiwara et al^19^ also found *PROX1* overexpression to suppress cell growth and tumor formation in HeLa cells, partially mediated by protein kinase C β. Additionally, it was also demonstrated that *PROX1* strongly inhibits the proliferation of neuroblastoma cell lines as well as cyclin D1, cyclin-A, and cyclin B1, consistent with a role in cell cycle arrest.^20^

In contrast, loss of *PROX1* function has been detected in hematologic malignancies, sporadic breast cancer, and carcinomas of the biliary system.^21–23^ Mutations and DNA methylation appear to be the major causes behind loss of *PROX1* function in some tumors.^22–24^ Recently, an antimetastatic role of *PROX1* was observed in *PROX1*-silenced hepatocarcinoma cell lines via *TWIST1* gene inhibition.^25^ In OSCC, Sasahira et al^26^ demonstrated that *PROX1* and *FOXC2* act as oncogenes by inducing lymphangiogenesis and angiogenesis. Additionally, *PROX1* was associated with tumor progression (pT and clinical stage), nodal metastasis, and lymphovessel density.^26^ These studies suggest that *PROX1* may function as an oncogene or a tumor suppressor gene in a cancer type-specific manner.

Interestingly, a previous microarray study done by our group revealed that *PROX1* transcripts were downregulated in OSCC when compared with tumor-free margins.^7,27^ However, the underlying mechanism by which *PROX1* acts in oral cancer is still unclear. In this study, we analyzed the expression levels of *PROX1* transcripts and proteins as well as *PROX1* amplification and methylation status in OSCC tissues and tumor-free surgical margins. We also investigated how *PROX1* affects cell proliferation, differentiation, survival, migration, and invasion in a squamous cell carcinoma cell line.

## METHODS

### Tumor Samples

Specimens were obtained by surgical resection from OSCC patients (men, ≥40 years old) admitted for diagnosis and treatment at the Arnaldo Vieira de Carvalho Cancer Institute, Heliópolis Hospital, and Hospital das Clínicas (School of Medicine, University of São Paulo, Brazil). Histopathological diagnosis was performed according to World Health Organization classification for tumors. Clinicopathological staging was determined by the TNM classification of the International Union Against Cancer.^28^ All patients have provided written informed consent to participate in this study that was approved by the Brazilian National Ethics Committee (Process #16491) and meets the Declaration of Helsinki.

Forty fresh surgical samples of primary OSCC and their corresponding nonneoplastic margin tissues were immediately snap-frozen in liquid nitrogen. After histological confirmation, all tissue samples were checked prior to RNA extraction so each OSCC sample contained at least 70% tumor cells. The corresponding surgical margins were reported as ‘tumor-free.’ The GENCAPO (Head and Neck Genome Project) consortium was responsible for collecting samples and initial processing, collecting clinical data, performing histopathological analysis, and obtaining informed consent from each patient.

### Cell Culture

The human OSCC cell lines SCC4, SCC9, and SCC25 were obtained from the American Type Culture Collection (ATCC, Manassas, VA) and cultured as recommended in 1:1 mixture of Dulbecco Modified Eagle medium (DMEM) and Ham F12 medium (DMEM/F12; Invitrogen, Carlsbad, CA) supplemented with 10% fetal bovine serum (FBS), 400 ng/mL hydrocortisone (Sigma-Aldrich, St. Louis, MO), 100 μg/mL penicillin, and 100 μg/mL streptomycin. Cell lines were authenticated using the AmpFLSTR Identifiler PCR Amplification kit (Life Technologies) at the Special Techniques Laboratory of the Israelita Albert Einstein Hospital (LATE-HIAE), São Paulo, Brazil. Cell line matched (100% identity) the STR profile described by the ATCC database.

### Plasmid DNA Transfection for *PROX1* Expression

SCC9 cells were transfected with pCMV6 empty vector (mock) or pCMV6-*PROX1* expression vector (OriGene, Rockville, MD), containing full-length human *PROX1* cDNA, using Lipofectamine 2000 (Invitrogen), following the manufacturer's instructions. Transfected cells were selected in medium with 300 μg/mL G418 (Invitrogen).

### Real-Time RT-PCR

Total RNA was extracted from cells using TRIzol (Invitrogen, Carlsbad, CA) according to the manufacturer's instructions. First-strand cDNAs were synthesized with a High Capacity cDNA Archive kit (Applied Biosystems, Grand Island, NY, USA). Quantitative RT-PCR (RT-qPCR) reactions were performed using an Applied Biosystems 7500 Real-Time PCR System with SYBR Green I Dye (Applied Biosystems) according to the manufacturer's instructions. The following primers were used to detect *PROX1* and endogenous “housekeeping” gene *HPRT*: *PROX1* (forward 5’-CTCCGTGGAACTCAGCGC-3’ and reverse 5’-GCCGGCTTAAGAGGGCTG-3’) and *HPRT* (forward 5’-CCACCACCCTGTTGCTGTA-3’ and reverse 5’-TCCCCTGTTGACTGGTCAT-3’).

The following primers were used to investigate genes identified as deregulated in oral cancer (data not shown): *GATA3* (forward 5’-CGTCCTGTGCGAACTGTCA-3’ and reverse 5’-GTCCCCATTGGCATTCCTCC-3’), *NOTCH1* (forward 5’-GGTGAACTGCTCTGAGGAGATC-3’ and reverse 5’-GGATTGCAGTCGTCCACGTTGA-3’), *E2F1* (forward 5’-CATCCCAGGTCACTTCTG-3’ and reverse 5’-GACAACAGCGGTTCTTGCTC-3’), and *WISP3* (forward 5’-ACTGTAGCCTGGAACCATTACT-3’ and reverse 5’-TGGTCACCCTGTTAGATATTCCC-3’).

All RT-qPCR reactions were performed in total volume of 25 μL, containing 1 μL of cDNA sample, 400 nM of each primer, 12.5 μL of SYBR Green Master Mix (Applied Biosystems). The PCR amplifications were performed under the following conditions: initial denaturation for 10 minutes at 95°C, followed by 40 cycles of 10 seconds at 95°C and 1 minute at 60°C. Melting curve analysis was performed at the end of each cycling to confirm amplification specificity and lack of primer dimer. Quantitative analysis was performed according to the Pfaffl mathematical model.^29^

The differential expression of *PROX1* in OSCC samples was divided into high and low according to the value obtained from RT-qPCR normalized with the respective tumor-free margins. The cut-off value was set up at the median expression level of OSCC samples.

### Gene Amplification Assay

Gene amplification analysis of the OSCC tissue and tumor-free margins was performed by qPCR using an Applied Biosystems 7500 Real-Time PCR System with SYBR Green I Dye. The following primers were used to detect *PROX1* (F: 5’-AGCCTCCGTGGAACTCAGC-3’ and R: 5’-CCACCAGCAGGAAAGAGAAA-3’) and *ZNF80* (F: 5’-CTGTGACCTGCAGCTCATCCT-3’ and R: 5’-TAAGTTCTCTGACGTTGACTGATGTG-3’). *ZNF80* was used as a reference gene to normalize the qPCR data.^30^ Next, both primer sets generated were validated in silico. Quantitative analysis was performed according to Terribas et al.^29,31^ Briefly, we calculated the ΔCq value for the difference between the unknown and calibrator samples. The relative quantity was calculated as RQ = E^-ΔCq^ and the normalized relative quantity (NRQ) as NRQ = RQ^PROX1^/RQ^ZNF80^. Further, we calculated relative copy number (RCN) as RCN = NRQ/RF, where RF is rescaling factor, which is the geometric mean of the NRQ values of a set of 17 normal oral mucosa bearing 2 *PROX1* copies. RCN values close to 1 indicate the presence of 2 *PROX1* copies and RCN values >1 indicate *PROX1* positive amplification.

### Methylation Assay

Methylation status of *PROX1* in OSCC tissue and tumor-free margins was evaluated by the EpiTect Methyl Custom qPCR Array technology according to the manufacturer's instructions (Qiagen, Valencia, CA). Thirty fresh OSCC samples and 17 corresponding nonneoplastic margins were treated with a simple DNA methylation-sensitive and methylation-dependent restriction enzyme digestion without bisulfite conversion. After digestion, the remaining DNA was quantified using DNA methylation real-time PCR arrays in an ABI 7500 Real-time system. Methylation status was expressed as percentage of inputted DNA that was methylated as determined using EpiTect Methyl DNA methylation PCR data analysis (Qiagen, http://www.sabiosciences.com/dna_methylation_data_ analysis.php), following the manufacturer's instructions. The methylated DNA status in the samples was divided into 2 groups based on the following cut-off values: >50% (hypermethylated) or <50% (hypomethylated).

### Immunohistochemistry

Immunohistochemical analysis of PROX1 protein expression was performed using the polymer-linked method in 30 OSCC samples and 8 nonneoplastic tissues. Four-μm-thick tissue sections were cut, deparaffinized, and subjected to antigen recovery treatment with 1 mM EDTA buffer target retrieval solution, pH 8.0 at 95°C, in a water bath for 20 minutes. Endogenous peroxidase activity was blocked by incubating with phosphate-buffered saline (PBS) and 3% hydrogen peroxidase for 30 minutes. After washing with PBS, the sections were treated with protein block (Dako, X0909, Carpinteria, CA) and then incubated with monoclonal rabbit anti-PROX1 (Abcam, clone 5G10, Cambridge, UK) diluted 1:50, followed by the Envision Dual Link System horseradish peroxidase (HRP) method (Dako, K4061). Reactions were initiated by incubating the sections with 3,3’-diaminobenzidine tetrahydrochloride (Dako, K3468). The negative controls were obtained by substituting the primary antibody with nonimmune serum. To quantify PROX1 protein expression in OSCC samples and nonneoplastic margins, immunohistochemistry (IHC) scores were used as described by Yamatoji et al,^32^ with minor modifications. Next, the percentage of PROX-1 positive epithelial cells was evaluated at x100 magnification throughout each entire section and scored as follows: 1, 0% to 25%; 2, 25% to 50%; 3, 25% to 75%; and 4, 75% to 100%. PROX1 immunoreaction intensity was also scored as 1, weak; 2, moderate; and 3, intense. The percentage of PROX1 positive tumor cells and staining intensity were then multiplied to obtain each PROX1 IHC score. We considered these samples positive when PROX1 IHC score was >7.37 (average score for nonneoplastic margins).

### Western Blotting

Cells were lysed in RIPA buffer (50 mM Tris–HCl pH 7.4, 150 mM NaCl, 1 mM EDTA, 1% NP-40, 1% deoxycholic acid, 0.5% sodium dodecyl sulfate, 1 mM phenylmethylsulfonyl fluoride, 1 mM N-ethylmaleimide, 1 mM dithiothreitol, 10 μg/mL soybean trypsin inhibitor, 1 μg/mL leupeptin, and 1 μg/mL aprotinin). We revealed 30 μg of total protein per sample using 8% sodium dodecyl sulfate (SDS) polyacrylamide gel electrophoresis. The primary antibody to detect PROX-1 (Abcam, clone 5G10), p21 (Abcam, ab7960), cyclin D1 (Santa Cruz, sc-20044, Dallas, Texas, USA) and actin (Sigma, clone CA-74) were incubated overnight at 4°C at the dilution 1:300. The nitrocellulose membranes were developed using a chemiluminescent Western blot system (Enhanced Chemiluminescent Western blot kit; GE Healthcare, Vienna, Austria).

### Bromodeoxyuridine Labeling Index

Cells were plated in chamber slides (30,000 cells/well) and serum starved for 48 hours. Next, cell culture medium supplemented with 10% FBS was added for 24 hours followed by bromodeoxyuridine reagent incubation for 2 hours at 37°C. Bromodeoxyuridine (BrdU) incorporation in proliferating cells was estimated using an immunohistochemical analysis kit (Invitrogen, BrdU Staining Kit). The BrdU-labeling index, expressed as the percentage of cells labeled with BrdU, was determined by counting 1000 cells in 3 independent reactions using the Kontron 400 image analysis system (Zeiss Axio Imager A1, Dublin, California, USA).

### Ki-67 Index

Cells were cultured as described above and fixed after incubation with culture medium supplemented with 10% FBS for 24 hours. Cells were incubated with monoclonal antibodies against Ki-67 (Clone MIB-5, Dako), followed by the Envision Dual Link System HRP (Dako). The Ki-67 index was calculated using an image analysis system by counting labeled nuclei of 1000 cells in 3 independent reactions, expressed as percentage of Ki-67-positive cells.

### Proliferation Curves

Cells were seeded in 24-well plates in DMEM/F12 containing 10% FBS and incubated in serum-free medium for 48 hours. After this period, DMEM/F12 10% FBS was added back and cells from triplicate wells were trypsinized after 24, 48, 72, 96, 120, and 144 hours and counted in the Neubauer chamber.

### Flow Cytometry

For cell cycle analysis, SCC9 cells were seeded in 100-mm dishes and serum starved for 48 hours. Next, the cell culture medium supplemented with 10% FBS was added and the cells were collected after 24 hours. Cells were fixed in cold 70% ethanol, stored at −20°C, washed in cold PBS, and treated with 10 mg/mL of RNase for 1 hour at 37°C. After staining with 50 μg/mL of propidium iodide for 2 hours at 4°C, cell distribution in the cell cycle was analyzed by flow cytometry.

Cells were analyzed on a FACSCalibur flow cytometer equipped with an argon laser (Becton-Dickinson, San Jose, CA). At least 10,000 events were analyzed in each sample. Quantitative flow cytometric analysis was performed using CellQuest software (Becton-Dickinson).

### Immunocytochemistry

Immunostaining was performed on cytokeratins (CK) 1, 10, 13, 14, 16, 18, and 19 using the Envision Dual Link System HRP (Dako, K4061). Cells were incubated with mouse anti-CK10 (1:100, DEK-10, BioGenex, Fremont, California, USA), anti-CK16 (1:300, ab8741, Abcam), anti-CK18 (1:1500, DC10, Dako), anti-CK19 (1:2000, RCK108, Dako), rabbit anti-CK1 (1:200, ab24643, Abcam), anti-CK13 (1:100, ab92551, Abcam), and anti-CK14 (1:2000, ab7800, Abcam) followed by the polymer-link method. The slides were scanned into high-resolution images using an Aperio ScanScope XT (Aperio Technology, Inc), Vista, CA). The images were then viewed in an Image Scope (Aperio Technology, Inc) and immunostaining was quantified using the Positive Pixel Count algorithm, v9 (Aperio).

### Apoptosis Analysis

Apoptosis index was determined by annexin V-FITC labeling. The cells were collected, washed with PBS, and resuspended in the binding buffer (10 mM HEPES pH 7.4, 150 mM NaCl, 5 mM KCl, 1 mM MgCl_2_, and 1.8 mM CaCl_2_) containing annexin V-FITC at 1:500. After 20 minutes incubation in the dark at room temperature, the cells were also stained with propidium iodide (PI, Sigma-Aldrich). Apoptosis was analyzed on a FACSCalibur flow cytometer equipped with an argon laser (Becton Dickinson) and quantified as the number of annexin V-FITC positive and PI negative cells divided by the total number of cells. At least 10,000 events were analyzed in each sample.

### Migration and Invasion Assays

The in vitro cell migration and invasion assays were performed in 24-well plates (Corning, Inc, New York, NY) using modified Boyden chamber inserts with a polycarbonate filter membrane containing 8-μm pores. For the invasion assay, the BD BioCoat Matrigel Invasion Chamber was used in 24-well plates (BD Biosciences). The cells (1 × 10^5^) were suspended in 200 μL of serum-free DMEM/F12 and seeded onto the upper compartment of the transwell chamber and DMEM/F12 containing 10% FBS was used in the lower chamber for stimulation. After 24 hours of incubation for the migration and invasion analysis, the medium in the upper chamber was removed, and the filters were fixed in 4% paraformaldehyde following 20 minutes in methanol 100%. The cells on the lower surface were stained with toluidine blue solution in 1% borax for 15 minutes. A cotton swab mechanically removed cells that did not migrate through the pores. The die was eluted using 1% SDS and the absorbance was measured at 560 nm. Two independent experiments were performed with triplicates.

### Statistical Analysis

To analyze *PROX1* mRNA expression levels in tissue samples and OSCC cell lines, we used Wilcoxon nonparametric test and one-way analysis of variance (ANOVA) with Tukey post-test, respectively. The different *PROX1* expression levels in tissue samples were divided into 2 groups (high vs low) and the median expression level was used as the cut-off value. We used one-way ANOVA with Tukey post-test to analyze proliferation of PROX1-overexpressing SCC9 and control cells, CK immunoexpression, and apoptosis index. We used Fisher exact test to estimate the statistical difference between *PROX1* gene expression levels and clinicopathological parameters such as mean age, tumor location, tumor size-pT, nodal metastasis-pN, pathological grade, lymphatic and/or perineural invasion (PNI), and recurrence. When clinical information was not described in the files, it was considered as missing data. Kaplan–Meier product-limit estimation with log-rank (*P* < 0.05) was used for survival analysis from lifetime data according to gene expression levels. The GraphPad Prism 5 statistical package (GraphPad Software, Inc, La Jolla, CA, USA) was used for statistical analysis.

## RESULTS

### *PROX1* Gene and Protein Expression is Downregulated in OSCC Compared With Normal Oral Mucosa

**FIGURE 1:**
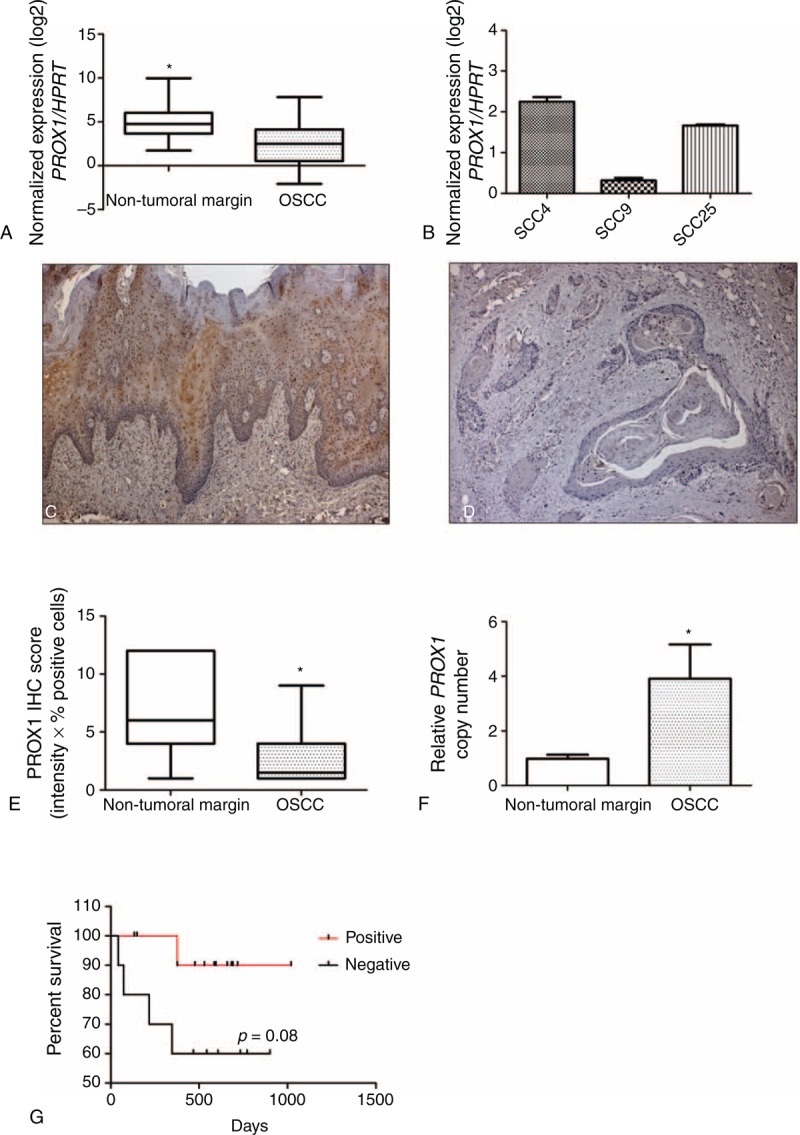
*PROX1* expression is downregulated in OSCC samples. Normalized expression of *PROX1* transcripts in nontumor margins and OSCC samples (A) and in OSCC cell lines (B). The full line corresponds to the median value for each group. Asterisk indicates statistically significant difference between OSCC and nontumor samples (*P* < 0.001, Wilcoxon) (A). SCC9 showed reduced *PROX1* mRNA expression levels (B). Immunohistochemical detection of PROX-1 in nontumoral margins (C) and OSCC (D). PROX-1 expression was found in the nucleus and cytoplasm of the epithelial cells located in suprabasal layers for the nontumor margin samples. The PROX-1 protein expression was significantly lower in OSCC samples than nontumoral margins (*P* < .001, Mann–Whitney *U* test). The IHC scores for nontumoral margins and OSCC range from 1.00 to 12.00 (median, 6.0) and from 1.00 to 9.00 (median, 1.50), respectively (E). Kaplan–Maier estimation of overall survival of OSCC patients according to *PROX1* amplification (*P* value according to log-rank test) (F). IHC = immunohistochemistry, OSCC = oral squamous cell carcinoma, PROX1 = prospero homeobox 1.

We performed RT-qPCR amplifications on 40 fresh surgical samples of primary OSCC and their corresponding nonneoplastic margin tissues and OSCC cell lines to assess the *PROX1* mRNA expression level. Significantly higher *PROX1* expression levels were found in nonneoplastic margins (Min–Max, 10.59–1010) compared with OSCC samples (Min–Max, 1.19–226; 5.17-fold, *P* < .001; Wilcoxon test) (Figure 1A). OSCC cell line SCC-9 exhibited low *PROX1* mRNA expression levels when compared with SCC-4 and SCC-25 cell lines (Man–Whitney, *P* < .001) (Figure 1B). When *PROX1* expression levels (higher vs lower) were correlated with clinicopathological features and disease outcome (Table 1), there was no significant association with age group, tumor location, pTNM classification, pathological grade, lymphatic and/or PNI, and local recurrence.

**TABLE 1:**
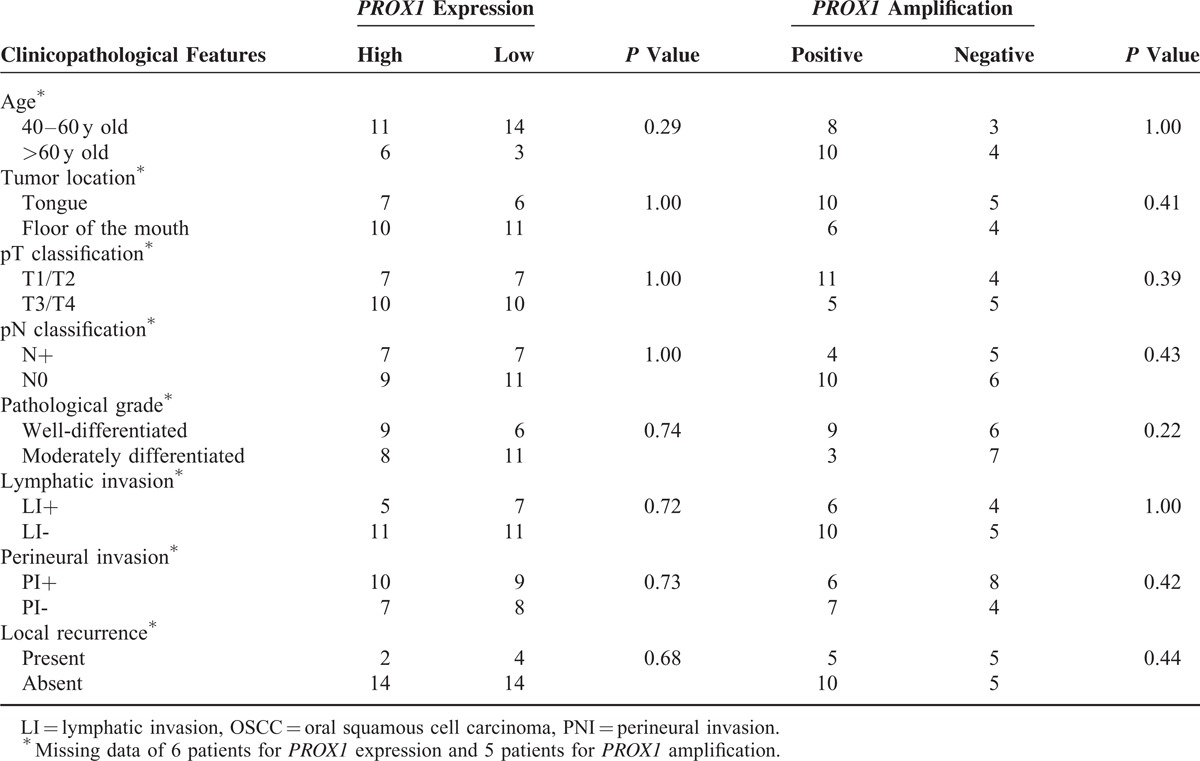
*PROX1* gene expression levels and amplification associated with OSCC clinicopathological features (*P* value Fisher exact test)

IHC was also performed to confirm lower PROX-1 expression in OSCC samples compared with nonneoplastic margins. Immunoreactivity for PROX1 was observed as a nuclear and cytoplasmatic expression, predominantly in suprabasal layers of nonneoplastic margins (Figure 1C). Only 3 OSCC samples were considered positive for PROX-1 expression after IHC score analysis. Similar to mRNA expression, OSCCs showed significantly lower PROX-1 immunoexpression than nonneoplastic margins (*P* < .001; Man–Whitney test) (Figure 1D and E). Although no statistical difference was found, well-differentiated OSCC showed high PROX1 expression in well-differentiated areas mostly represented by keratin pearls.

### *PROX1* Amplification, but not Methylation Status, Is Associated With Better Survival Rate

To investigate a possible mechanism for *PROX1* downregulation, methylation analysis was performed on 30 fresh OSCC samples. No difference between OSCC samples and normal oral tissues (data not shown) was found because only 4 tumors and 2 margins presented 50% mean frequency of promoter methylation. *PROX1* amplification was seen in 16/23 OSCC samples. There was a significant difference in *PROX1* amplification between OSCC and tumor-free margin samples (*P* < .001, Figure 1F).

*PROX1* amplification and methylation showed no significant association with clinicopathological features such as tumor location, pTNM classification, pathological grade, and lymphatic and/or PNI (Table 1). However, the *P* value for the survival curve, determined by the log-rank test, was significantly different in the survival rates between positive and negative *PROX1* amplification groups (*P* = 0.08, log-rank). Patients with positive *PROX1* amplification had much more favorable prognosis than those with negative amplification (Figure 1G). *PROX1* methylation status was unrelated to overall survival (*P* = 0.34, log-rank). We also performed a correlation analysis between *PROX1* expression and amplification and found no significant difference. However, there was a positive correlation (*r* = 0.02, *P* = 0.90; Spearman correlation), suggesting that *PROX1* expression tends to be higher in tumors with *PROX1* amplification (data not shown).

### *PROX1* Overexpression Promotes an Antiproliferative Phenotype in OSCC Cells

**FIGURE 2:**
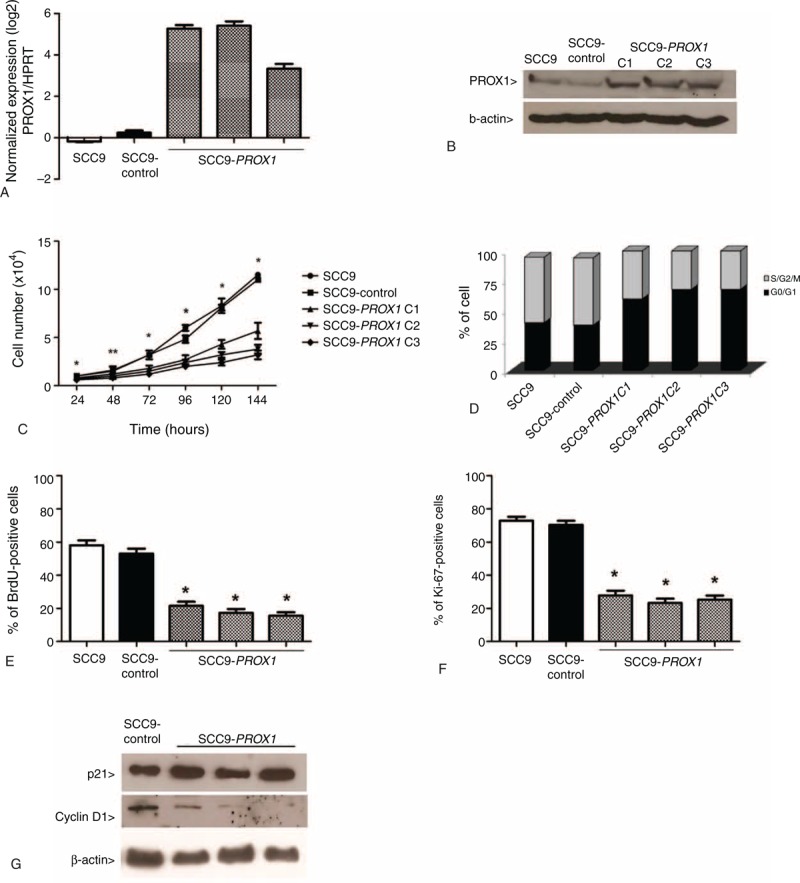
*PROX1* overexpression inhibits cell proliferation. *PROX1* mRNA expression levels by qRT-PCR in SCC9 cells, SCC9-control, and 3 constitutively *PROX1*-expressing cell clones (A). Representative Western blot analysis in the cell lines previously described. The β-actin was shown as an internal control (B). *PROX1* overexpression was observed in the mRNA and protein levels. Proliferation curves (C), cell cycle analysis by flow cytometry (D), assay measuring BrdU (E), and Ki-67 expression (F) demonstrate that *PROX1*-overexpressing cells have statistically decreased proliferation compared with control cells (*P* < 0.001). Western blot for expression of p21 and cyclin D1 (G). *PROX1*-overexpressing cells showed a decrease in cyclin D1 expression compared with SCC9 and SCC9-control. BrdU = Bromodeoxyuridine, PROX1 = prospero homeobox 1.

To determine whether *PROX1* overexpression contributes to proliferation, differentiation, apoptosis, migration, and invasion of OSCC cells, *PROX1* was stably expressed in SCC9 cell line because it showed the lowest *PROX1* mRNA endogenous levels. *PROX1* overexpression was confirmed by increased expression of both *PROX1* mRNA and protein in SCC9-*PROX1* clones but not in the control (SCC9-control, Figure 2A and B). Three stable *PROX1*-overexpressing clones and 1 control clone were chosen for further analysis.

SCC9-*PROX1* cells proliferation significantly decreased when compared with SCC9 and SCC9-control, as assessed by proliferation curves (*P* = .04), BrdU incorporation (*P* < .001), and Ki-67 expression (*P* < .001) (Figure 2C, E, and F). SCC9-*PROX1* cell clones also showed increased G0-G1 population in cell cycle analysis (Figure 2D). Also, a decrease in cyclin D1 protein expression was observed when compared with SCC9- control cells (Figure 2G). There was no significant difference in p21 expression in SCC9-*PROX1* and control cells (data not shown).

**FIGURE 3:**
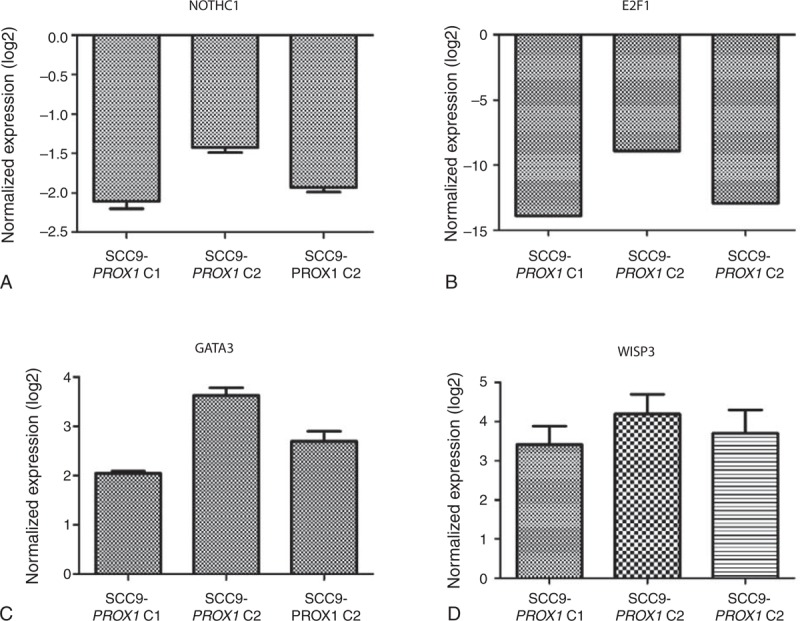
*PROX1* transcriptionally regulates the expression of *NOTCH1*, *E2F1*, *GATA3*, and *WISP3*. *NOTCH1* (A) and *E2F1* (B) were downregulated in SCC9-PROX1 cell clones compared with control cells as well as *GATA3* (C) and *WISP3* (D) that were upregulated. PROX1 = prospero homeobox 1.

Genes related to cell proliferation were selected arbitrarily from a previous microarray analysis (unpublished data). qRT-PCR was used to investigate selected genes related to cell proliferation: *GATA3* and *WISP3* were upregulated (2.79 and 3.77 fold-change mean, respectively), whereas *E2F1* and*NOTCH1* were downregulated (-11.90 and -1.82 fold-change mean, respectively) in SCC9-PROX1 cell clones compared with SCC9-control (Figure 3).

To further characterize the effects of *PROX1* overexpression, in vitro assays to measure apoptosis, migration, and invasion were carried out. *PROX1* overexpression did not significantly modulate those events when compared with control cell (Supplemental file 1, 2, and 3, http://links.lww.com/MD/A82).

### *PROX1* Overexpression Reduces CK Expression Associated With Poorly Differentiated OSCC

**FIGURE 4:**
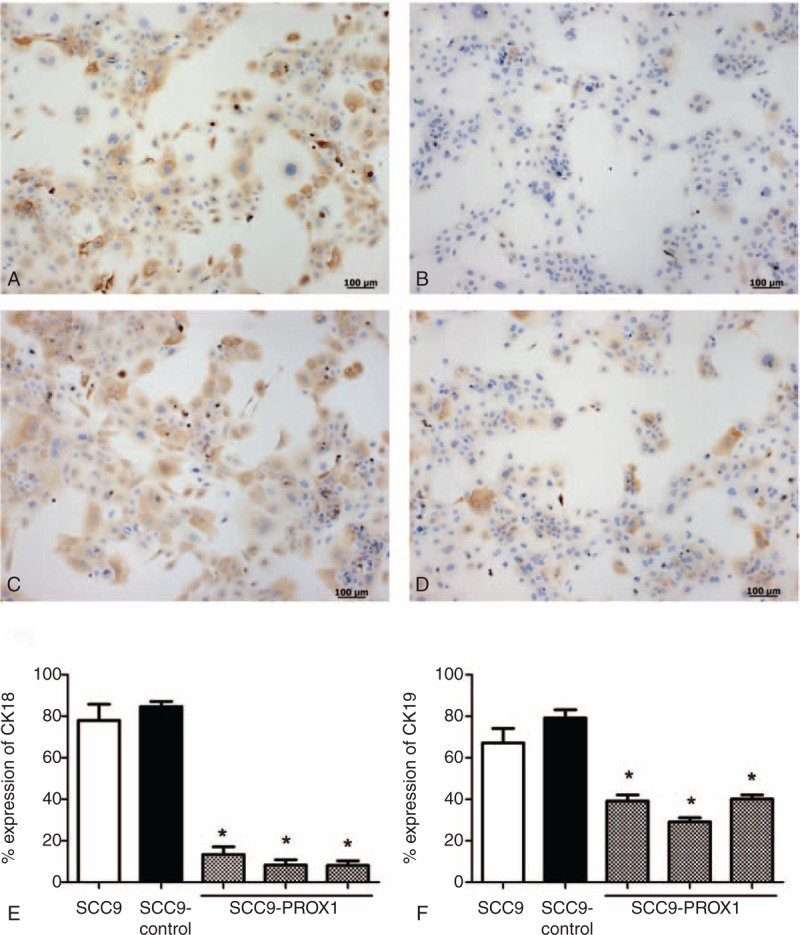
*PROX1* overexpression reduces CK18 and CK19 expression in SCC9 cell line. Immunoexpression of CK18 and 19 in SCC9 and SCC9 control (A and C) and in SCC9-*PROX1* cell clones (B and D), respectively. SCC9 and SCC9-control cells showed an intense expression of CK18 (A) and CK19 (C) around the nucleus. SCC9-*PROX1* cell clones revealed significantly reduced CK18 (B) and CK19 (D) protein expression compared with SCC9 and SCC9-control (*P* < 0.001)(E and F). PROX1 = prospero homeobox 1.

To assess the effects of *PROX1* overexpression on OSCC differentiation, immunocytochemical analysis on CKs 1, 10, 13, 14, 16, 18, and 19 were performed. Immunocytochemical evaluation showed that all CK reactivity was homogeneously restricted to the cytoplasm of positive cells. There was discrete reduced expression for CK1 and 13 in SCC9-*PROX1* cells compared with the control cells (data not shown). CK18 and 19 expression were significantly reduced in SCC9-*PROX1* cell clones when compared with SCC9 and SCC9 control cells (*P* < .001, one-way ANOVA, Figure 4). Expression of CK10, 14, and 16 were expressed in almost all cells with no observed difference between SCC9-*PROX1* cell clones and control cells (data not shown).

## DISCUSSION

*PROX1* was previously reported by us as downregulated in OSCC after microarray analysis.^7^ This prompted us to further investigate *PROX1* role in this neoplasm. *PROX1* mRNA expression levels was found lower in OSCC samples when compared with their nontumoral margins. Its protein expression levels were also decreased in OSCC samples when compared with nontumoral margins, suggesting that loss of *PROX1* expression in OSCC is associated with a malignant phenotype. Additionally, only well-differentiated tumors showed on IHC a weak positivity for PROX-1protein. In agreement with these findings, in vitro overexpression of *PROX1* was associated with reduced cell proliferation of OSCC cells. Also, a more favorable prognosis was seen in patients with positive *PROX1* amplification. All these findings favor the hypotheses that *PROX1* may act as a tumor suppressor gene in OSCC.

*PROX1* is a homeobox gene that plays an essential role in early development, controlling critical pathways for proliferation and differentiation.^14^ This gene has been identified as a master gene of lymphangiogenesis and is also involved in cell fate decisions in the central nervous system,^33^ liver, and pancreas.^12,34^*PROX1* may act as a tumor suppressor gene or oncogene based on the fact that Prox1 affects differentially the expression of genes that promote or inhibit proliferation and cell cycle progression.^35^ In some cancer types as breast and hepatocarcinoma, *PROX1* is inactivated because of neoplastic transformation and progression of cancer cells,^18,19,36^ indicating a tumor suppressor function.^36^ Moreover, *PROX1* plays a tumor-promoting role in colon cancer, malignant astrocyticglioma, and kaposiform hemangioendothelioma.^15,16,37^

As mentioned above, our findings suggest that decreased or loss of *PROX1* expression is evident after epithelial malignant transformation. Consistent with this finding, *PROX1* amplification is seen in patients with a better prognosis, although *PROX1* expression was not related with clinical features presented by our patients, probably because of the low number of cases. Interestingly, Sasahira et al^26^ found *PROX1* overexpression in OSCC samples, which was associated with local progression, clinical stage lymphovessel density, nodal metastasis, and worse prognosis. These opposite findings in the same histologic tumor may be because of different patient profiles, and also regional differences between populations. It is well known that OSSC tumors may have different behavior when considering patient gender, age, localization, and etiological factors.^27^ Therefore, future studies confirming the clinical and prognostic value of *PROX1* in OSCC should consider evaluating a large cohort of patients with the same clinical profile.

Decreased expression of *PROX1* was also found in other types of tumors as lymphomas, sporadic breast cancer, hematological malignancies, and carcinomas of the liver, biliary duct, and pancreatic biliary system.^22–24,38,39^ In hepatocellular carcinomas, reduced *PROX1* expression is associated with poorly differentiated tumors and worst prognosis.^18^ This study did not find association of *PROX1* expression levels with prognosis and survival in OSCC.

Epigenetic silencing is one of the mechanisms responsible for *PROX1* inactivation in tumors.^22,23^ Hypermethylation of CpG islands was identified as a mechanism for *PROX1* inactivation in breast carcinomas and carcinomas of the biliary system.^22,23^ This encouraged us to investigate if *PROX1*-decreased expression in OSCC was related to promoter DNA methylation. We performed methylation assays in 30 OSCC and 17 nontumoral margins, and there were no significant differences in promoter methylation between the samples. However, it is a common knowledge that gene expression is a complex process with multiple levels of control, and besides methylation, homeobox genes are also under microRNA and translational regulation control; for example, genetic alterations could also be an alternative mechanism leading to *PROX1* downregulation in OSCC. These genetic alterations have been previously found in carcinomas of the biliary system, cell lines derived from hematological malignancies, and hepatocellular carcinomas.^18,21,22^

We investigated *PROX1* amplification in OSCC and nontumoral margins because gene amplification can lead to increased gene expression levels. It was observed that OSCC samples showed higher gene copy number when compared with nontumoral margins. To investigate if tumors with higher *PROX1* expression levels also exhibited *PROX1* amplification, we performed correlation analysis between amplification and expression in the same tumor samples. Although we found no significant differences, the positive correlation suggests that *PROX1* expression tends to be higher in tumors with *PROX1* amplification. Neither methylation nor amplification analysis was associated with clinicopathological features such as tumor location, pTNM classification, pathological grade, and lymphatic and/or PNI. We hypothesized that other genetic and epigenetic mechanisms may be involved in *PROX1* expression regulation, leading to its downregulation. Interestingly, patients with positive *PROX1* amplification had much more favorable prognosis than those with negative amplification, suggesting that *PROX1* amplification may be associated with high *PROX1* expression levels, contributing to better prognosis of OSCC.

To assess the biological effects of *PROX1* in OSCC, we stably expressed the *PROX1* gene in the SCC9 cell line, which had reduced expression levels of *PROX1* mRNAs and proteins. The results presented here reveal that *PROX1* overexpression inhibits in vitro cell proliferation of OSCC, which corroborates with previous results in other tumor cells from esophageal squamous cell carcinoma and hepatocellular carcinoma.^18,40^ Under normal development, this gene has been associated with the regulation of cell proliferation by downregulating cell cycle inhibitors *CDKN1B* (p27) and *CDKN1A* (p21).^13^ In this study, *PROX1* overexpression reduced cyclin D1 expression, without interfering with p53 and p21 expression. Overexpression of cyclin D1 produces shorter G1 phase and less dependency on growth factors, resulting in abnormal proliferation.^41^ Foskolou et al^20^ recently demonstrated that *PROX1* was sufficient to decrease Cdc25A and induce p27-Kip1 but not p21-Cip1 or p53, negatively regulating neuroblastoma carcinogenesis. Additionally, *PROX1* also suppresses proliferation of hepatocarcinoma cells via inhibiting Twist to trigger p53-dependent senescence.^36^ Possibly *PROX1* regulates different cell cycle proteins, which could account for the context dependent function of this gene in cancer pathogenesis. We believed that *PROX1* could contribute to reduce the proliferation of OSCC-altering cyclin D1 expression.

We also found that *PROX1* transcriptionally regulates the expression of genes related to cell proliferation, as demonstrated by upregulated genes *WISP3* and *GATA3* and downregulated genes *NOTCH1* and *E2F1* in SCC9-*PROX1* cells. *WISP3* is downregulated in aggressive cancers and lost in most invasive carcinomas, including breast carcinoma,^42^ where it is tumor-inhibitory and seems to suppress breast tumor growth and invasiveness of tumor cells.^43,44^ Loss of *GATA3* expression was found to be a reliable indicator of poor prognosis in breast cancer,^45^ and lack of *GATA3* function is associated with lower survival, more malignant histological attributes, metastasis, and increased tumor mass.^46^*E2F1* plays a pivotal role in regulating the expression of genes involved in G1-S transition and DNA synthesis, and it is the most well-known transcription factor regulated by the cyclin/Cdk/Rb pathway.^47^*E2F1* regulatory binding sites are also present in the *PROX1* gene promoter. Additionally, *E2F1* is overexpressed in OSCC and silencing of E2F-1 inhibits proliferation and induces apoptosis.^48,49^ Thus, *PROX1* may act in altering *E2F1* expression and downregulating cyclin D1, resulting in reduced cell proliferation in *PROX1* overexpressing cells.

CKs are structural components of the epithelial cytoskeleton and constitute major proteins for cellular differentiation. Alterations in the expression pattern of some specific CKs have been reported to contribute to progression of OSCC.^50,51^*PROX1* overexpressing cells showed significantly reduced expression of CK18 and CK19. These CKs were previously associated with worse prognosis, poorer pathologic differentiation grade, tumor recurrence, and metastasis to lymph nodes in OSCC.^50–52^ Thus, the *PROX1* gene may contribute in the differentiation of OSCC by downregulating these CKs because differentiated cells did not express CK18 and CK19. Interestingly, *PROX1* also contributes to tumor differentiation in hepatocellular carcinomas and pancreatic carcinoma.^38^

In summary, our results provide evidence that *PROX1* is differentially expressed between nontumoral margins and OSCC, its overexpression reduces proliferative activity and contributes to the differentiation of OSCC cells, and its amplification is related to better prognosis of OSCC samples, suggesting that the downregulation of this gene in oral cancer contributes to the malignant phenotype.

## References

[1] Argiris A Karamouzis MV Raben D Head and neck cancer. *Lancet* 2008; 371:1695–1709. 18486742 10.1016/S0140-6736(08)60728-X PMC7720415

[2] Scully C Bagan J Oral squamous cell carcinoma overview. *Oral Oncol* 2009; 45:301–308. 19249237 10.1016/j.oraloncology.2009.01.004

[3] Abate-Shen C Deregulated homeobox gene expression in cancer: cause or consequence? *Nat Rev Cancer* 2002; 2:777–785. 12360280 10.1038/nrc907

[4] Hassan NM Hamada J Murai T Aberrant expression of HOX genes in oral dysplasia and squamous cell carcinoma tissues. *Oncol Res* 2006; 16:217–224. 17294802 10.3727/000000006783981080

[5] Zhu F Li J Li WX Overexpression and clinicopathological significance of homeobox gene Quox-1 in oral squamous cell carcinoma. *J Biochem Mol Biol* 2004; 37:671–675. 15607025 10.5483/bmbrep.2004.37.6.671

[6] Destro MFSS Bitu CC Zecchin KG Overexpression of HOXB7 homeobox gene in oral cancer induces cellular proliferation and is associated with poor prognosis. *Int J Oncol* 2010; 36:141–149. 19956843

[7] Rodini CO Xavier FC Paiva KB Homeobox gene expression profile indicates HOXA5 as a candidate prognostic marker in oral squamous cell carcinoma. *Int J Oncol* 2012; 40:1180–1188. 22227861 10.3892/ijo.2011.1321 PMC3584618

[8] Bitu CC Destro MF Carrera M HOXA1 is overexpressed in oral squamous cell carcinomas and its expression is correlated with poor prognosis. *BMC Cancer* 2012; 12:146. 22498108 10.1186/1471-2407-12-146 PMC3351375

[9] Wigle JT Harvey N Detmar M An essential role for Prox1 in the induction of the lymphatic endothelial cell phenotype. *EMBO J* 2002; 21:1505–1513. 11927535 10.1093/emboj/21.7.1505 PMC125938

[10] Vaessin H Grell E Wolff E Prospero is expressed in neuronal precursors and encodes a nuclear protein that is involved in the control of axonal outgrowth in *Drosophila*. *Cell* 1991; 67:941–953. 1720353 10.1016/0092-8674(91)90367-8

[11] Wigle JT Oliver G Prox1 function is required for the development of the murine lymphatic system. *Cell* 1999; 98:769–778. 10499794 10.1016/s0092-8674(00)81511-1

[12] Sosa-Pineda B Wigle JT Oliver G Hepatocyte migration during liver development requires Prox1. *Nat Genet* 2000; 25:254–255. 10888866 10.1038/76996

[13] Wigle JT Chowdhury K Gruss P Prox1 function is crucial for mouse lens-fibre elongation. *Nat Genet* 1999; 21:318–322. 10080188 10.1038/6844

[14] Elsir T Smits A Lindstrom MS Transcription factor PROX1: its role in development and cancer. *Cancer Metastasis Rev* 2012; 31:793–805. 22733308 10.1007/s10555-012-9390-8

[15] Dadras SS Skrzypek A Nguyen L Prox-1 promotes invasion of kaposiform hemangioendotheliomas. *J Invest Dermatol* 2008; 128:2798–2806. 18580962 10.1038/jid.2008.176

[16] Petrova TV Nykanen A Norrmen C Transcription factor PROX1 induces colon cancer progression by promoting the transition from benign to highly dysplastic phenotype. *Cancer cell* 2008; 13:407–419. 18455124 10.1016/j.ccr.2008.02.020

[17] Elsir T Qu M Berntsson SG PROX1 is a predictor of survival for gliomas WHO grade II. *Br J Cancer* 2011; 104:1747–1754. 21559010 10.1038/bjc.2011.162 PMC3111172

[18] Shimoda M Takahashi M Yoshimoto T A homeobox protein, PROX1, is involved in the differentiation, proliferation, and prognosis in hepatocellular carcinoma. *Clin Cancer Res* 2006; 12 (20 pt 1):6005–6011. 17062673 10.1158/1078-0432.CCR-06-0712

[19] Hagiwara K Ito H Murate T PROX1 overexpression inhibits protein kinase C beta II transcription through promoter DNA methylation. *Genes Chromosomes Cancer* 2012; 51:1024–1036. 22833470 10.1002/gcc.21985

[20] Foskolou IP Stellas D Rozani I PROX1 suppresses the proliferation of neuroblastoma cells via a dual action in p27-Kip1 and Cdc25A. *Oncogene* 2013; 32:947–960. 22508481 10.1038/onc.2012.129

[21] Nagai H Li Y Hatano S Mutations and aberrant DNA methylation of the PROX1 gene in hematologic malignancies. *Genes Chromosomes Cancer* 2003; 38:13–21. 12874782 10.1002/gcc.10248

[22] Laerm A Helmbold P Goldberg M Prospero-related homeobox 1 (PROX1) is frequently inactivated by genomic deletions and epigenetic silencing in carcinomas of the bilary system. *J Hepatol* 2007; 46:89–97. 17069925 10.1016/j.jhep.2006.07.033

[23] Versmold B Felsberg J Mikeska T Epigenetic silencing of the candidate tumor suppressor gene PROX1 in sporadic breast cancer. *Int J Cancer* 2007; 121:547–554. 17415710 10.1002/ijc.22705

[24] Nagai MA Ros N Bessa SA Differentially expressed genes and estrogen receptor status in breast cancer. *Int J Oncol* 2003; 23:1425–1430. 14532986

[25] Chang TM Hung WC Transcriptional repression of TWIST1 gene by Prospero-related homeobox 1 inhibits invasiveness of hepatocellular carcinoma cells. *FEBS Lett* 2012; 586:3746–3752. 22982861 10.1016/j.febslet.2012.08.034

[26] Sasahira T Ueda N Yamamoto K PROX1 and FOXC2 act as regulators of lymphangiogenesis and angiogenesis in oral squamous cell carcinoma. *PloS One* 2014; 9:e92534. 24647631 10.1371/journal.pone.0092534 PMC3960274

[27] Severino P Alvares AM Michaluart P Jr Global gene expression profiling of oral cavity cancers suggests molecular heterogeneity within anatomic subsites. *BMC Res Notes* 2008; 1:113. 19014556 10.1186/1756-0500-1-113 PMC2632665

[28] Greene FL Sobin LH A worldwide approach to the TNM staging system: collaborative efforts of the AJCC and UICC. *J Surg Oncol* 2009; 99:269–272. 19170124 10.1002/jso.21237

[29] Pfaffl MW A new mathematical model for relative quantification in real-time RT-PCR. *Nucleic Acids Res* 2001; 29:e45. 11328886 10.1093/nar/29.9.e45 PMC55695

[30] D’Haene B Vandesompele J Hellemans J Accurate and objective copy number profiling using real-time quantitative PCR. *Methods* 2010; 50:262–270. 20060046 10.1016/j.ymeth.2009.12.007

[31] Terribas E Garcia-Linares C Lazaro C Probe-based quantitative PCR assay for detecting constitutional and somatic deletions in the NF1 gene: application to genetic testing and tumor analysis. *Clin Chem* 2013; 59:928–937. 23386700 10.1373/clinchem.2012.194217

[32] Yamatoji M Kasamatsu A Yamano Y State of homeobox A10 expression as a putative prognostic marker for oral squamous cell carcinoma. *Oncol Rep* 2010; 23:61–67. 19956865

[33] Lavado A Oliver G PROX1 expression patterns in the developing and adult murine brain. *Dev Dyn* 2007; 236:518–524. 17117441 10.1002/dvdy.21024

[34] Burke Z Oliver G PROX1 is an early specific marker for the developing liver and pancreas in the mammalian foregut endoderm. *Mech Dev* 2002; 118:147–155. 12351178 10.1016/s0925-4773(02)00240-x

[35] Li L Vaessin H Pan-neural Prospero terminates cell proliferation during *Drosophila* neurogenesis. *Genes Dev* 2000; 14:147–151. 10652268 PMC316344

[36] Chang TM Hung WC The homeobox transcription factor PROX1 inhibits proliferation of hepatocellular carcinoma cells by inducing p53-dependent senescence-like phenotype. *Cancer Biol Ther* 2013; 14:222–229. 23291986 10.4161/cbt.23293 PMC3595304

[37] Elsir T Eriksson A Orrego A Expression of PROX1 is a common feature of high-grade malignant astrocytic gliomas. *J Neuropathol Exp Neurol* 2010; 69:129–138. 20084020 10.1097/NEN.0b013e3181ca4767

[38] Schneider M Buchler P Giese N Role of lymphangiogenesis and lymphangiogenic factors during pancreatic cancer progression and lymphatic spread. *Int J Oncol* 2006; 28:883–890. 16525637

[39] Takahashi M Yoshimoto T Shimoda M Loss of function of the candidate tumor suppressor PROX1 by RNA mutation in human cancer cells. *Neoplasia* 2006; 8:1003–1010. 17217617 10.1593/neo.06595 PMC1783717

[40] Akagami M Kawada K Kubo H Transcriptional factor PROX1 plays an essential role in the antiproliferative action of interferon-gamma in esophageal cancer cells. *Ann Surg Oncol* 2011; 18:3868–3877. 21452064 10.1245/s10434-011-1683-6

[41] Todd R Hinds PW Munger K Cell cycle dysregulation in oral cancer. *Crit Rev Oral Biol Med* 2002; 13:51–61. 12097237 10.1177/154411130201300106

[42] Huang W Zhang Y Varambally S Inhibition of CCN6 (Wnt-1-induced signaling protein 3) downregulates E-cadherin in the breast epithelium through induction of snail and ZEB1. *Am J Pathol* 2008; 172:893–904. 18321996 10.2353/ajpath.2008.070899 PMC2276413

[43] Huang W Gonzalez ME Toy KA Blockade of CCN6 (WISP3) activates growth factor-independent survival and resistance to anoikis in human mammary epithelial cells. *Cancer Res* 2010; 70:3340–3350. 20395207 10.1158/0008-5472.CAN-09-4225 PMC2856127

[44] Pal A Huang W Li X CCN6 modulates BMP signaling via the Smad-independent TAK1/p38 pathway, acting to suppress metastasis of breast cancer. *Cancer Res* 2012; 72:4818–4828. 22805309 10.1158/0008-5472.CAN-12-0154 PMC3506182

[45] Chou J Provot S Werb Z GATA3 in development and cancer differentiation: cells GATA have it!. *J Cell Physiol* 2010; 222:42–49. 19798694 10.1002/jcp.21943 PMC2915440

[46] Mehra R Varambally S Ding L Identification of GATA3 as a breast cancer prognostic marker by global gene expression meta-analysis. *Cancer Res* 2005; 65:11259–11264. 16357129 10.1158/0008-5472.CAN-05-2495

[47] Chen HZ Tsai SY Leone G Emerging roles of E2Fs in cancer: an exit from cell cycle control. *Nat Rev Cancer* 2009; 9:785–797. 19851314 10.1038/nrc2696 PMC3616489

[48] Du Y Zhang S Wang Z Induction of apoptosis and cell cycle arrest by NS398 in oral squamous cell carcinoma cells via downregulation of E2 promoter-binding factor-1. *Oncol Rep* 2008; 20:605–611. 18695912

[49] Yuan H Jiang F Wang R Lentivirus-mediated RNA interference of E2F-1 suppresses Tca8113 cell proliferation. *Mol Med Rep* 2012; 5:420–426. 22076063 10.3892/mmr.2011.668

[50] Zhong LP Chen WT Zhang CP Increased CK19 expression correlated with pathologic differentiation grade and prognosis in oral squamous cell carcinoma patients. *Oral Surg Oral Med Oral Pathol Oral Radiol Endod* 2007; 104:377–384. 17095259 10.1016/j.tripleo.2006.07.019

[51] Mikami T Cheng J Maruyama S Emergence of keratin 17 vs. loss of keratin 13: their reciprocal immunohistochemical profiles in oral carcinoma in situ. *Oral Oncol* 2011; 47:497–503. 21489858 10.1016/j.oraloncology.2011.03.015

[52] Fillies T Werkmeister R Packeisen J Cytokeratin 8/18 expression indicates a poor prognosis in squamous cell carcinomas of the oral cavity. *BMC Cancer* 2006; 6:10. 16412231 10.1186/1471-2407-6-10 PMC1379654

